# General practice workforce retention strategies: an umbrella review

**DOI:** 10.1186/s12875-026-03446-8

**Published:** 2026-07-15

**Authors:** L. Jefferson, S. Golder, A. Castro-Avila, E. Webster, H. Anderson

**Affiliations:** 1 Centre for Primary Care & Health Services Research, University of Manchester, Manchester, UK; 2 Department of Health Sciences, University of York, York, UK; 3 Academic Foundation Doctor, York Teaching Hospital, York, UK

**Keywords:** Career Mobility, General Practice, General Practitioners, Nurse Practitioners, Primary Health Care, Systematic Review

## Abstract

**Objective:**

We summarise international evidence of interventions to support retention of the general practice workforce, mapping evidence by country, method and workforce group.

**Design:**

Umbrella overview of reviews. We mapped evidence according to occupational group, intervention type and outcome to identify trends and gaps. Varied study designs were included, though only reviews with evaluations of workforce interventions were included. Exploratory studies describing factors associated with retention were excluded.

**Setting:**

International primary care and general practice.

**Participants:**

General practice workforce, with studies grouped by general practitioners, nurses working in general practice and mixed health professional groups.

**Main outcome measures:**

Workforce retention, including length of employment, stability rates, settlement rates, vacancy rates, unfilled positions, turnover numbers or rates, attrition rates, intentions to stay, leave and return.

**Results:**

Of the 4,919 articles identified through database searching (Medline, Embase, PsycInfo and Medrxiv 6th February 2025) and citation tracking, we included five reviews. The majority of evidence was concentrated in Australia and the US and there was a lack of policy evaluations in some fields. While stronger evidence exists for interventions such as professional development and fostering supportive environments, mixed findings were reported on financial incentives and limited formal evaluations were found for some intervention types, and professional groups.

**Conclusion:**

This overview of research underscores the need for more research evaluating interventions to support the general practice workforce, particularly across wider international settings.

## Background

Imbalance in healthcare workforce capacity is challenging general practice workforces internationally. In the UK, rising patient demand due to aging populations and backlogs of unmet need generated during the COVID-19 pandemic [1, 2] has not been sufficiently met with workforce increases. The number of patients registered with practices in the England has risen by 14% since 2015 [3] and modelling suggests an additional 6,500 full time general practitioners (GPs) will be needed to meet demand by 2031 [4]. Meanwhile, GP participation rates, measured as full time equivalents (FTE), are decreasing (from 0.80 to 0.69 median FTE since 2015) [5] and around two thirds of GPs in England are considering leaving the profession within five years [6–8]. Internationally, pooled estimates from a meta-analysis of studies reporting turnover intentions suggest that 47% of GPs have a moderate intention to leave practice (CIs 39–55%) [9].

Research has reported increases in burnout and mental health problems driven by rising patient demand [1, 2], toxic workplace cultures, and poor work-life balance, which have been directly linked to intentions to leave [10, 11]. Across the UK health system as a whole, record rates of sickness absence also impact participation rates – in August 2021 almost 2 million sickness days were lost across the NHS in one month alone, with 560,000 of these due to mental health problems [12].

Questions exist around the future of the general practice model if trends continue [13, 14]. Practices are closing in some parts of the country, leaving patients without a local GP and resulting in increased workload for remaining practices; often in deprived areas where health needs are greatest [15, 16]. Fewer GPs are now taking on roles as GP partners – 10% fewer over the past 5 years [14] – fuelling practice closures and resulting in higher workloads and risk for remaining GP partners [14, 17]. GPs also report increasing attractiveness of ‘portfolio roles’ that are associated with fewer clinical hours in order to manage challenging workloads [11, 12, 18].

To solve workforce capacity issues, policy approaches can be grouped into one of the 3 Rs: encouraging either Recruitment, Retention or Return of the workforce. In general practice, recruitment interventions dominate international research, particularly focused on underserved or rural areas [19]. These strategies are an expensive, and long-term solution – at a cost of nearly £500,000 [20] and taking nearly ten years to train a fully-qualified GP in the UK. As a result, a shift has been seen towards retention strategies [21, 22]. The Department of Health and Social Care now recognise ‘shaping and supporting the workforce’ as one of three Areas of Research Interest outlined by the UK Department of Health and Social Care [23]. Developing strategies to support and retain this workforce is needed to meet the quadruple aim of healthcare, giving new emphasis on provider wellbeing alongside previously recognised aims of improving patient experience, population health and reducing healthcare costs. [24] This is welcome, not least because doctor wellbeing is important in its own right, but also since it effects the quality of patient care [25, 26].

In 2023, the Royal College of General Practitioners stressed a need for a comprehensive review of existing retention initiatives to support future policy and research. [14] Our umbrella review aims to meet this need through a summary of the international evidence of interventions to support retention of the general practice workforce. Specifically, we map evidence by country, approach and workforce group to enable insights for future research and policy.

## Methods

We conducted our umbrella review according to the Cochrane guidelines for systematic reviews [27] followed the PRIOR guideline for reporting the review [28]. All search strategies were developed by an Information Specialist (SG) and peer reviewed. We searched databases (Medline, Embase, PsycInfo and Medrxiv) and Google Scholar for reviews of general practice workforce retention conducted from 2010 to 6th February 2025. To focus our review on empirical evidence evaluating interventions, we limited our searches to include only systematic reviews and scoping reviews, using the criteria below. Full searches are available in Appendix 1.

### Inclusion criteria

#### Type of participants

Studies in the general practice workplace setting were considered, including doctors, nurses and other primary care health professionals. Trainee groups were excluded. International terminology was considered, for example the use of ‘family practice’ in the USA. Owing to the large number of reviews in hospital care settings, only reviews with a focus on general practice or primary care settings were included. Reviews focusing *only* on incentivising rural practice retention were excluded since they addressed slightly different aims (retention to a location rather than broader workforce retention). These also tended to be based in countries with particular issues of rurality; such as Australia, Canada and USA, which may not be transferable to other contexts.

#### Type of intervention

Interventions at either an individual, practice or system level were included. Reviews were excluded if there was no evaluation of an intervention i.e. if they were exploratory in nature.

#### Type of outcome measures

Measures of workforce retention, turnover or future intentions were included (for example, length of employment, stability rates, settlement rates, vacancy rates, unfilled positions, turnover numbers or rates, attrition rates, intention to stay/leave, intentions to return).

#### Types of study design

Review methodologies were included, including systematic reviews, meta-analyses, scoping reviews and reviews of reviews.

No language limits were placed on the search. As well as reference checking all the included studies, we conducted forward citation searches using CitationChaser. Reviews of multiple health sector groups were excluded, owing to their tendency to not report outcomes separately for general practice workers.

### Selection of studies

We used the software tool Covidence [29] to conduct screening, firstly of titles and abstracts, and later screening full texts of articles deemed potentially relevant. Both stages were undertaken by two independent reviewers (LJ, SG, AC, EW, HA or VD), with arbitration by a third reviewer where necessary. Excluded articles and reasons for exclusion were used to generate a flow diagram as recommended by the PRISMA statement [30].

### Data extraction

Data extraction was performed by two reviewers (AC, LJ) using a pre-piloted data extraction form, with a 20% sample cross-checked and inter-rater reliability assessed. Information was extracted regarding country, number of included studies, proportion of studies with focus on rurality, search dates, health professional group, nature and direction of outcomes. To enable a more detailed discussion of the evidence base, we extracted data from individual studies included in reviews, applying the inclusion criteria to describe only relevant included studies.

### Methodological quality

We assessed the quality of included systematic reviews using AMSTAR 2, with one reviewer undertaking assessment and second checking by a second reviewer. To assess study overlap across the included reviews, we calculated the corrected covered area (CCA):

$$CCA=\frac{N-r}{\left(rc\right)-r}$$

where N is the number of times publications appeared in reviews, r is the number of rows and c is the number of columns. We additionally used the following classification: 0–5% = slight; 6–10% = moderate; 11–15% = high; > 15% = very high overlap.

## Data synthesis

**Fig. 1:**
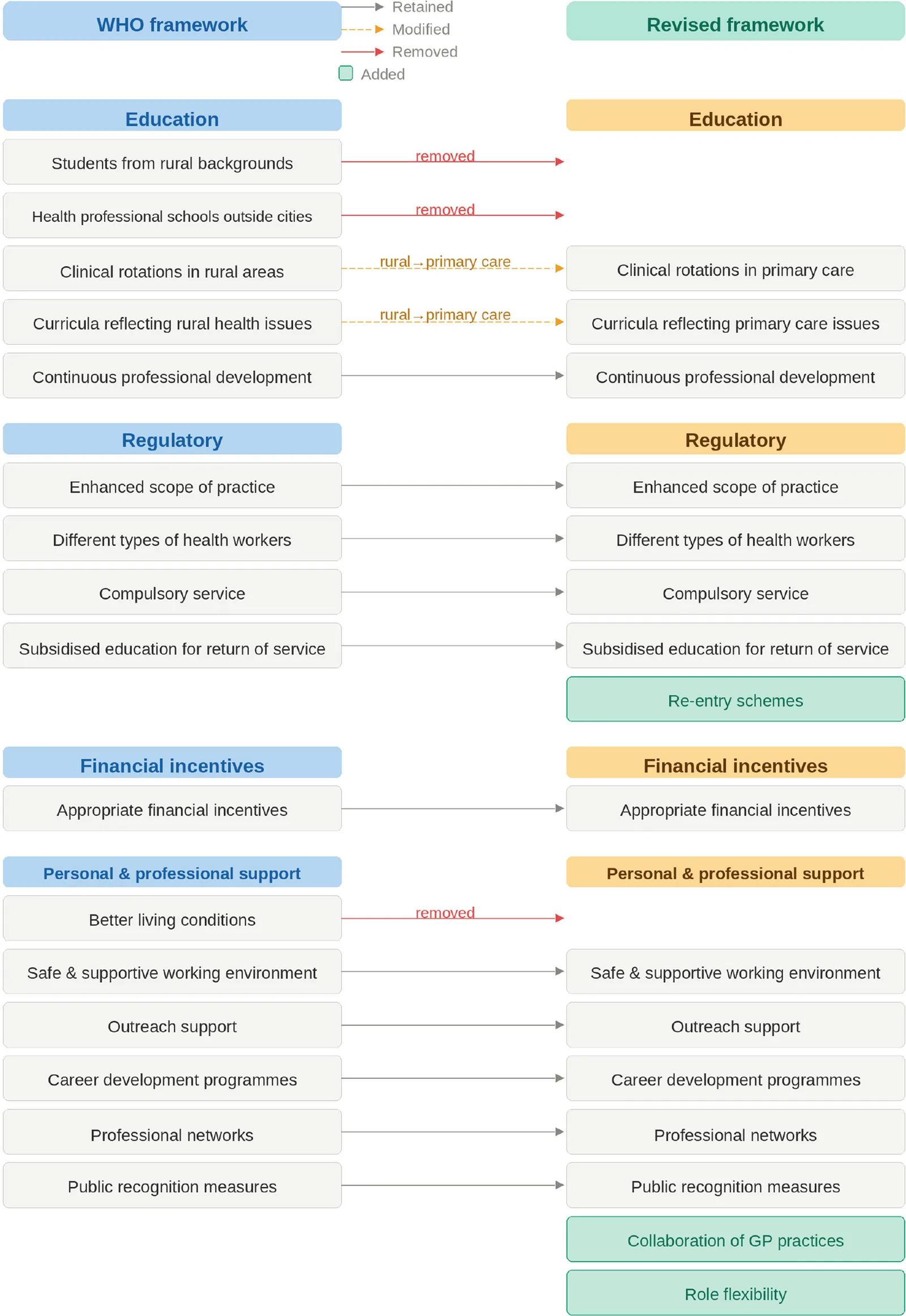
Adapted intervention framework

We used evidence mapping according to country, outcomes, professional groups and nature of intervention to outline evidence gaps. We categorised interventions as either: 1) Educational interventions, 2) Financial interventions, 3) Regulatory strategies or 4) Personal and professional support strategies using a framework previously used by the World Health Organisation [31] and a Cochrane review [32]. We adapted the framework (Fig. 1) to remove categories solely relevant to rural settings (since these were excluded), and expanded some subcategories to reflect additional interventions identified relating to re-entry schemes, role flexibility and collaboration between GP practices.

## Results

### Search results

**Fig. 2:**
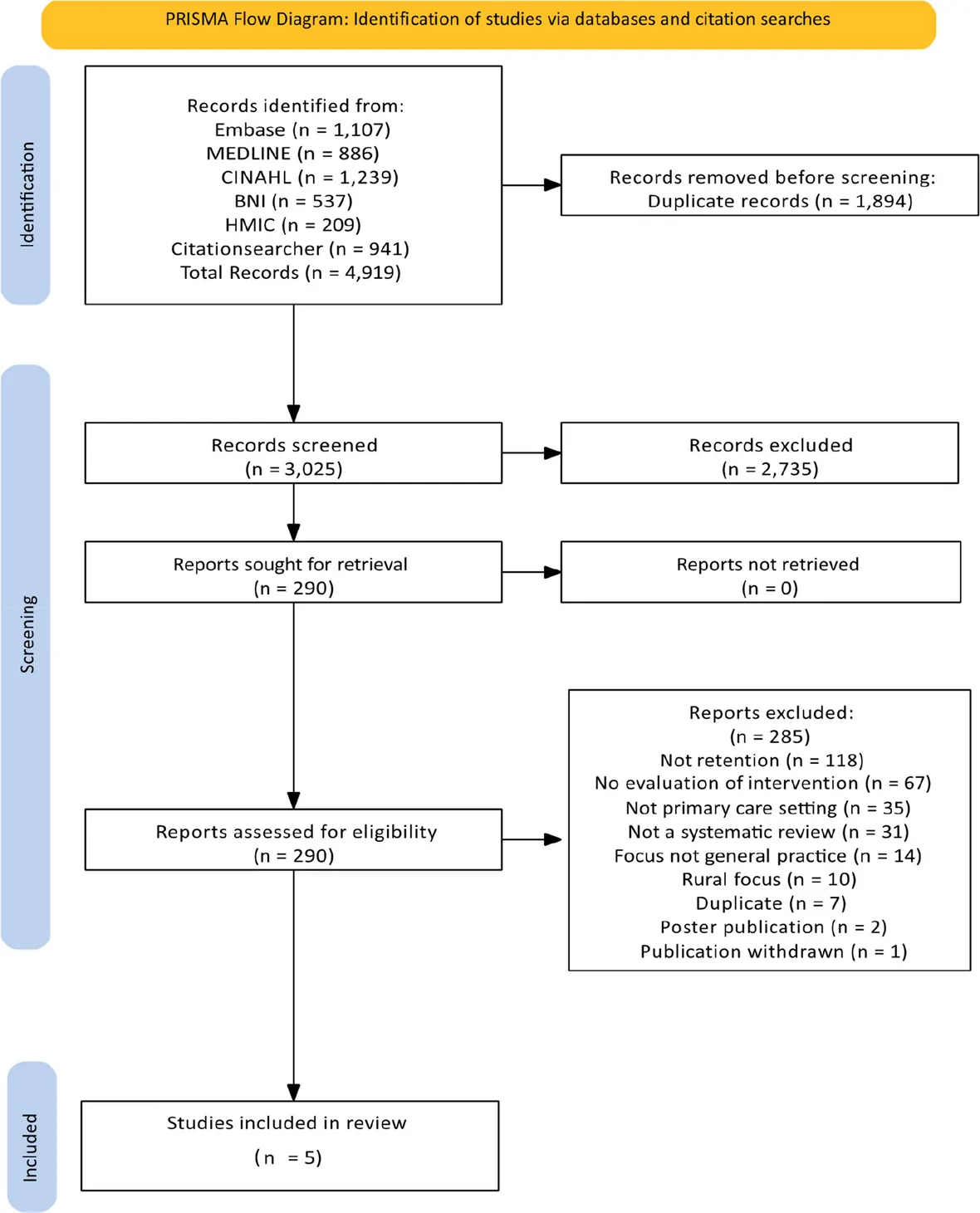
PRISMA diagram

We identified 4,919 articles through database searching and citation tracking. After removing duplicates, we identified 3,025 articles that were screened as titles and abstracts. At this stage, we excluded 2,735, leaving 290 for full text review. In total, five reviews [33–37] were identified that met our inclusion criteria (Fig. 2).

While retention was the focus of our review, it was often a secondary outcome in included studies. For example, policy interventions identified in Pettigrew’s review of working at scale tended to focus on patient outcome measures, rather than workforce outcomes [37]. A review by Harrington et al. [38] explored the use of mentoring in nurse practitioner programmes but no evaluations were identified, so this review was excluded from our search. Reviews by Peckham et al. [19], later updated by Mitchell et al., [39] also did not find any formal evaluations of GP retention strategies, instead providing summaries of studies exploring factors associated with retention. These reviews were excluded from this umbrella review.

### Study characteristics

Three reviews were focused on the nursing workforce in primary care settings [33–35] and two with a focus on GPs or primary care doctors [36, 37]. No included reviews explored interventions to support mixed professional groups working in general practice. Studies included in the reviews were undertaken between 1974 and 2019; the most recent search date of included reviews was 2022 (Table 1).

**Table 1:**
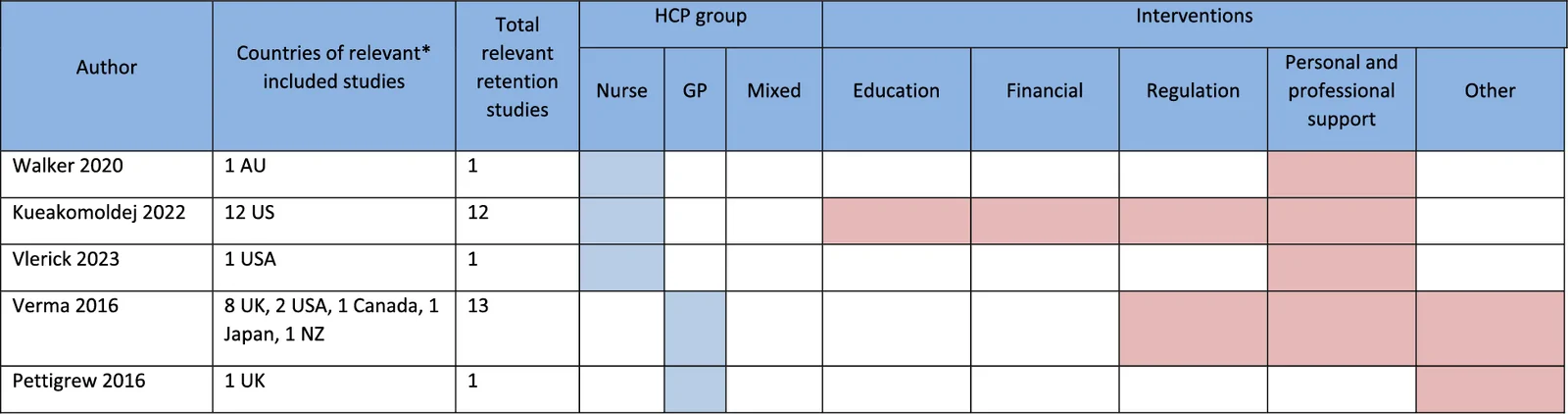
Principal characteristics of reviews and findings from retention studies according to intervention groups

*Abbreviations*: *AU* Australia, *UK* United Kingdom, *US* United States, *NZ* New Zealand

*Some reviews included wider workforce interventions not focused on retention. Numbers represent retention studies

**Fig. 3:**
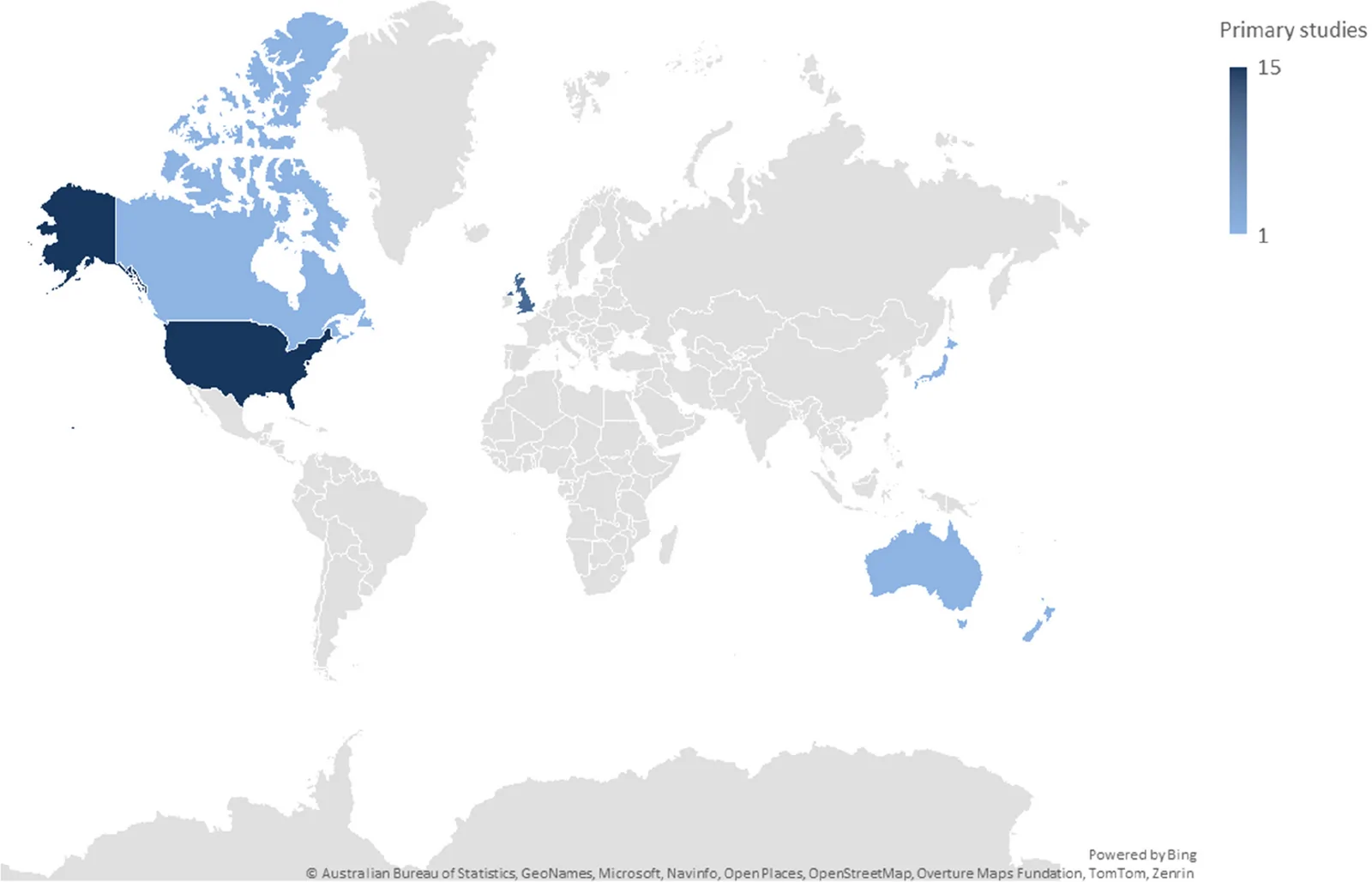
Locations of primary studies from included reviews

Figure 3 demonstrates the geographical spread of primary studies of the included reviews; highlighting studies predominantly from UK, North America and Australia. This is likely a result of the search strategies for included reviews being limited to these geographies.

### Quality assessment

For all five included reviews, the overall confidence in the results was deemed as critically low because they had two or more critical flaws. None of the included reviews conducted a meta-analysis or included a list of excluded studies providing an individual reason for exclusion. Our review found no overlap between included studies (CCA: 0%), highlighting the variation in searching across reviews due to different focus on health care professional groups and settings [Location for Table 2].

**Table 2:** AMSTAR 2 summary rating of the included reviews

Author, year	PICO	A priori methods	Study design selection	Search strategy	Study selection	Data extraction	Excluded studies	Included studies	RoB assessment	Funding sources	Statistical methods	Impact of RoB	Results interpretation accounts for RoB	Heterogeneity	Publication bias	Conflict of interest	Overall confidence in review results
Vlerick, 2023 [33]	Yes	Partial Yes	No	Partial Yes	Yes	No	No	Yes	Partial Yes	No	N/A	N/A	No	Yes	N/A	Yes	Critically low
Pettigrew, 2016 [37]	No	No	No	Partial Yes	No	No	No	Partial Yes	No	No	N/A	N/A	Yes	Yes	N/A	No	Critically low
Kueakomoldej, 2022 [35]	Yes	No	Yes	Partial Yes	Yes	Yes	No	Partial Yes	No	No	N/A	N/A	No	Yes	N/A	No	Critically low
Walker, 2020 [34]	No	No	Yes	Partial Yes	No	No	No	No	No	No	N/A	N/A	No	No	N/A	Yes	Critically low
Verma, 2016 [36]	Yes	No	No	Yes	Yes	Yes	No	Partial Yes	Yes	No	N/A	N/A	Yes	Yes	N/A	Yes	Critically low

### Evidence mapping

Our heatmaps by intervention type and outcome highlight potential evidence gaps in strategies to support general practice workforce retention. Across all included reviews and studies, a total of 40 interventions were identified (Table 3), with the majority reported as positive (n = 32). When disaggregated by occupational group (Table 4), 14 interventions related specifically to GPs and 26 to nurses. Outcomes were generally more consistently positive for nurses (n = 24 positive, 2 mixed) than for GPs (n = 8 positive, 5 mixed, 1 negative).

**Table 3:**
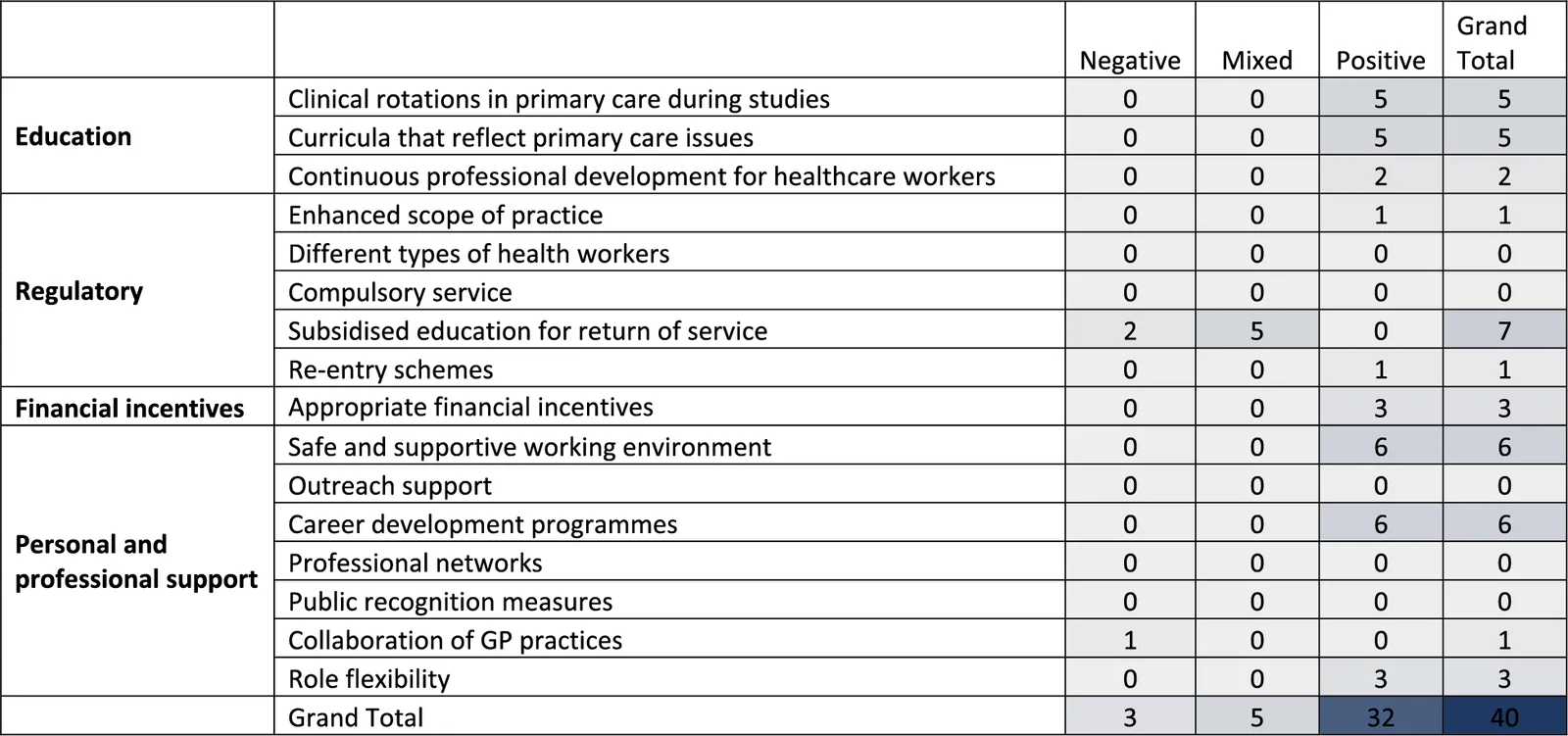
Heatmap of interventions by outcome, all reviews

**Table 4:**
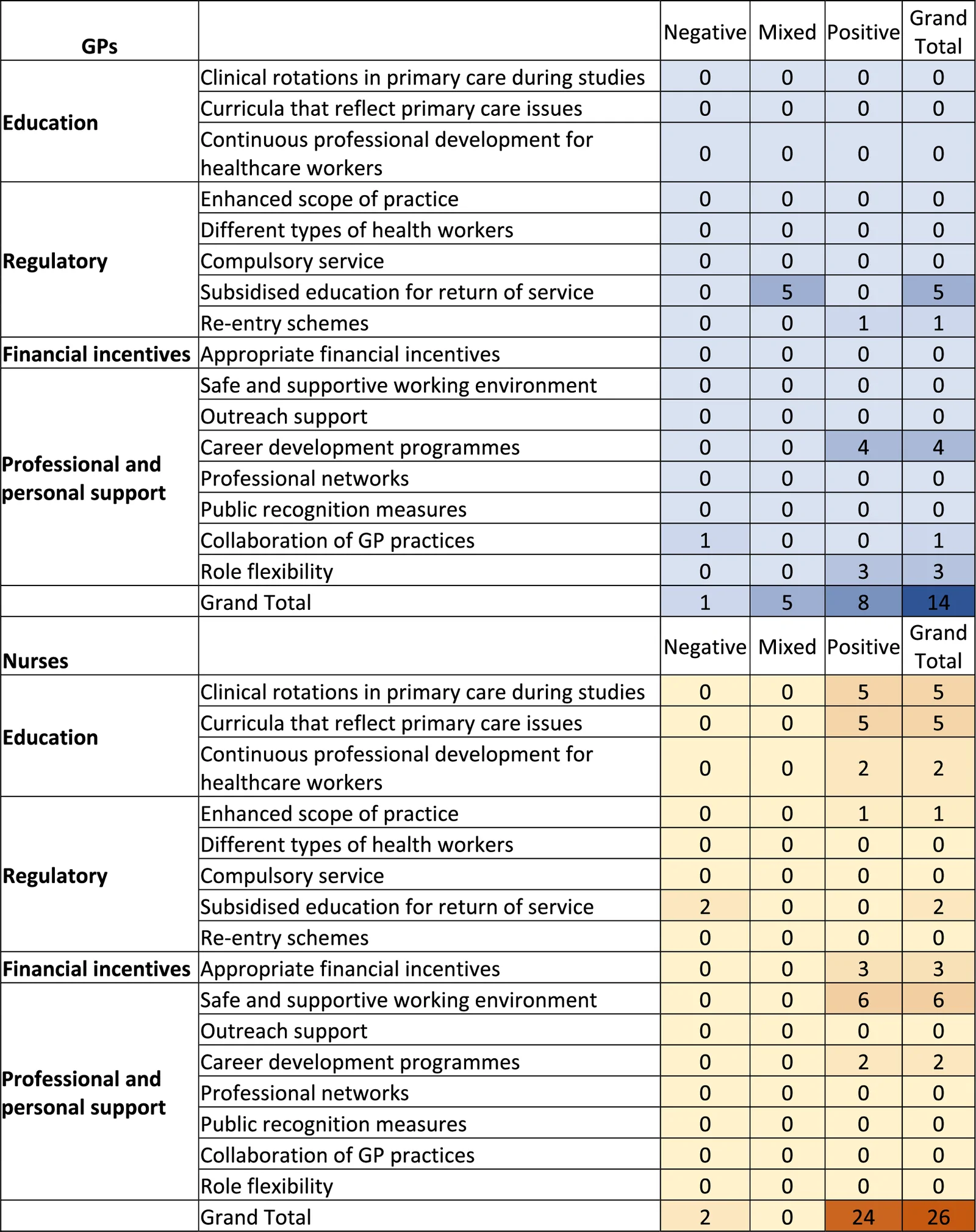
Heatmap of interventions by outcome and occupational group

Only one review found a potentially *negative* effect of interventions when considering retention. Pettigrew et al. [40] explored the use of working at scale in UK general practice and reported potential negative impact on retention. However, this was based on the evidence from one qualitative study.

#### Education

Educational interventions focused primarily on support for students or trainees, included rotations in primary care, curricula that reflect primary care issues and also providing continuous professional development (CPD) once qualified. Educational strategies were one of the most commonly reported approaches, although only captured in one review of nursing professionals. Positive outcomes were reported for clinical rotations in primary care (5 studies), curricula reflecting primary care issues (5 studies) and CPD (2 studies). However, among the studies looking at clinical rotations in primary care and curricula reflecting primary care issues, only one study had a comparison group [41–45]. The remaining studies measured number of students that worked in underserved primary care areas after graduation or factors contributing to their decision to work in primary care. In contrast, studies that assessed continuous development programmes focused on loan repayment schemes that included a strong professional development focus and factors making them successful at retaining staff in underserved communities [46, 47]. We found no studies evaluating educational interventions in GPs or allied health professionals.

#### Regulatory

From our included reviews, we found no studies exploring the impact of employing different types of health workers on retention outcomes, nor compulsory service, which is a strategy more often applied to retain rural health workers. One study with nurses found that being able to practice to the full scope of their profession influenced their decision to stay working in underserved primary care areas after completing a ‘National Health Service Corps’ programme [48]. Subsidised education for return of service produced mixed outcomes amongst GPs (5 studies). Three studies that had a comparison group found positive results, but they all assessed different programmes [49–51]. Matsumoto et al. [49] examined a Japanese nine-year mandatory service agreement where the candidate received fully funded undergraduate medical training in exchange for service in their home region. Prefectures with a relative shortage of physicians had higher settlement rates, and participants choosing primary care were more likely to settle (1.80 [95% CI 1.35–2.39]) [47]. Mathews et al. (2013) evaluated two return-for-service schemes in Canada (i.e., Family Medicine Bursary and Special Funded Residency Position), finding that doctors who received funding for their education were 3.2 times less likely to leave an underserved area during the repayment period. Pathman et al. (2004) evaluated five separate support-for-service financial incentives finding that retention rates were statistically significantly higher for obligated versus non-obligated doctors (Hazard ratio 0.70, 95% CI 0.51 to 0.96). Two further studies did not include a comparison group, therefore it is difficult to draw conclusions. One study reported on a postgraduate voluntary bonding scheme in New Zealand which recruited trainees to hard-to-staff communities for five years, with repayments starting after the third year. They found that 89% of graduates had opted out of the scheme three years after entering. Meanwhile, Nichols et al. (1977) explored an Arizona programme that subsidised out-of-state medical education by covering the difference between resident and non-resident tuition and offering incentives to medical schools to accept Arizona students. They report 62% of participants repaid their loan through in-state service.

Studies including nurses found negative results (2 studies). Pathman et al. (2012a) surveyed participants in the National Health Service Corps (NHSC) programme, finding clinicians motivated mostly by the financial support were less likely to have intentions to stay in their service sites over time. Negrusa et al. [52] found that NHSC participants were less likely to work in health professional shortage areas than non-participants in programme one year after completion (82% vs 95%).

One included review identified a study with positive retention outcomes in an evaluation of re-entry schemes for GPs [53]. The scheme provided support through tutorials and mentoring to 14 participants. Two participants had return to work after 6 months, while 11 had taken “positive steps” to return or had increased their working hours. In comparison, only one participant from the control group had made plans to return to general practice (denominator not reported).

#### Financial incentives

Three studies [46–48] found that competitive salary and productivity incentives were associated with intention to stay among nurses participating in loan repayment schemes in the US (i.e., NHSC and Obligatory Health Care Recruitment Program in Delaware). No studies were included reporting the use of financial incentives for GPs.

#### Professional and personal support interventions

Interventions that provided professional and personal support for both nursing and GP professionals were identified – all with positive retention outcomes. For GPs, evaluations of career development programmes identified positive outcomes in four studies [54–57]and four specifically identified role flexibility as supporting GP retention [53, 58–60]. As aforementioned, one review reported negative potential consequences of GP practices working at scale [40].

For nurses, interventions that created safe and supportive working environments influenced retention and intention to stay (6 studies). These findings came from two qualitative studies [61, 62], and four studies without a comparison group assessing loan repayment schemes (e.g., Obligatory Health Care Recruitment Program in Delaware and the NHSC) [46–48, 63].

Career development programmes (2 studies) were also associated with better retention of nurses in underserved communities. For example, Aysola et al. (2015) found that health centres with career ladder programmes had higher retention rates than centres without those programmes (39.4% vs. 21.2%, p = 0.0001). Similarly, nurses who belong to a practice-based research network had longer mean employment years in underserved areas than non-members (7.0 ± 6.8 years versus 4.0 ± 5.0 years, *p* = 0.003) [64]. Other strategies identified by the WHO framework of interventions [31] we used, such as outreach support, professional networks and public recognition measures lacked supporting evidence.

#### Summary

In summary, interventions addressing education and professional support appear most consistently beneficial, particularly for nurses, while career development programmes and role flexibility show promise for GPs. We found no evidence assessing compulsory service, inclusion of different health care workers in primary care, belonging to professional networks or public recognition measures. This evidence comes mainly from the US, the UK, Canada, Australia, New Zealand and Japan.

## Discussion

### Summary of findings

This review maps and synthesises the evidence base from existing reviews exploring strategies to support workforce retention in primary care settings. There are notable gaps, both in terms of when and where the reviews and included studies were undertaken. The most recent review used searches in 2022 and there was, in general, a lack of coverage of countries outside Australia, the US and the UK. Based on our critical appraisal of the included reviews, our overall confidence in the results was critically low due to two or more critical flaws. Further, the evidence base overall was dominated by observational and qualitative studies, with few comparison groups, which is surprising given this review focused on interventions.

Our evidence mapping reveals a general paucity of evidence across many intervention types. There were more studies from the included reviews exploring strategies to support nursing workforce outcomes and these tended to be consistently favourable, while for GPs there were fewer studies and more mixed results. Educational and professional support interventions showed the most consistent benefits, particularly for nurses. Educational strategies such as primary care rotations, relevant curricula and continuing professional development were associated with improved retention, though evidence for GPs was lacking. Career development, mentoring, role flexibility and supportive work environments were consistently linked to better retention for both groups.

Regulatory and financial approaches produced mixed results: some return-of-service schemes improved GP retention, while others involving nurses, showed negative effects. Evidence from one qualitative study [37] found a negative impact of working at scale on GP retention, though further research is now needed. Significant evidence gaps remain for other areas identified in the WHO framework we applied [31], including compulsory service, workforce mix, living conditions, professional networks and public recognition measures.

Across reviews, we found that workforce retention often featured as a secondary outcome, with many interventions primarily targeting patient outcomes or broader organisational goals. Meanwhile, a review of nurse practitioner mentoring^52^ and reviews of GP retention [19, 39] highlight a lack of formal evaluations, emphasising the need for more focused research in these areas.

### Implications for practice, policy, and future research

As per Cochrane and GRADE guidelines [27, 65], we urge caution in making firm recommendations for policy and practice due to the general lack of comparison groups, heterogeneous outcomes and contexts of included studies. However, when considered in the context of ongoing workforce challenges in UK general practice, some tentative implications can be drawn. Interventions identified in this umbrella review, particularly those focused on improving working conditions, professional support and career development, are consistent with international workforce strategies and recommendations that explicitly prioritise building supportive environments over single, short-term interventions [66]. They also align with findings from recent UK-based research, including a realist review which highlights the importance of relationships, human connection building in general practice, as well as learning and development and psychological safety [67]. Similarly, our analysis of national UK data on GP turnover suggests that positive workplace relationships and a sense of belonging are associated with improved retention [68].

Nevertheless, the lack of overlap between included studies (CCA: 0%) highlights the need for an updated evidence synthesis to build on these reviews, including varied primary care workforce groups and a specific focus on retention. This may highlight whether evidence gaps have been filled and allow a comprehensive assessment of the quality of included studies, since our focus here has been on evaluating the quality of included reviews. Broader global contexts also need to be understood – searches from included reviews were limited by geography.

### Strengths and limitations

This umbrella review provides a valuable insight into the existing evidence base, with this approach offering a high-level overview of the research gaps for interventions to support general practice workforce retention. The transferability of these findings is limited by important contextual differences across health systems. While challenges such as workload pressures, burnout and turnover intentions are widely reported, the scale and nature of shortages vary between countries, with some facing overall supply issues and others more localised maldistribution. Primary care workforce composition also differs, with varying use of multidisciplinary teams that may mitigate or redistribute pressures on doctors. In addition, differences in funding models, employment arrangements and the role of private sector provision may influence retention dynamics, including the potential for movement between sectors. As a result, interventions identified in one setting may not be directly applicable elsewhere without adaptation, highlighting the need for context-specific evidence.

Nevertheless, this umbrella review highlights evidence gaps that may help to prioritise future research efforts and policies to support future workforce retention. In addition to the geographical limitations of the searches of included review, there were also challenges in terms of how well evidence was reported in some studies. For example, it was common for reviews to report findings for recruitment and retention together, but often challenging to disentangle the evidence due to reporting.

## Conclusion

This research identifies significant gaps in the evidence base in this field and underscores the need for more targeted workforce retention research.

## Data Availability

The data used during the current study are available from the corresponding author on reasonable request.

## Appendix 1

### Search strategy

**Table 5:** Summary of inclusion and exclusion criteria

Inclusion	Exclusion
Primary care workforce. Using UK terminology such as general practitioners or international terminology such as ‘family practice’ or ‘family physician.’	Reviews of medical specialities outside primary care (acute or hospital medicine), owing to their tendency to not report outcomes separately for primary care workers
Interventions at either an individual, practice or system level	
Measures of workforce retention and intentions to leave practice were included (e.g.: length of employment, stability rates, settlement rates, vacancy rates, unfilled positions, turnover numbers or rates, attrition rates, intention to stay/leave, intentions to return)	Reviews not measuring retention (e.g. those solely exploring recruitment factors or patient outcomes)
Review methodologies (including systematic reviews, literature reviews or ‘evidence syntheses’, meta-analyses, scoping reviews and umbrella reviews)	Primary studies were excluded. Reviews not providing summaries of evaluations of interventions were excluded
Published after 01.01.2010	Published prior to 2010
All languages	

### Database Search Strategies

Ovid MEDLINE(R) ALL < 1946 to February 05, 2025 >

**Table:**

#	Search terms	Records
1	(systematic review or meta-analysis).pt	369935
2	meta-analysis/or systematic review/or systematic reviews as topic/or meta-analysis as topic/or "meta analysis (topic)"/or "systematic review (topic)"/or exp technology assessment, biomedical/or network meta-analysis/	412810
3	((systematic* adj3 (review* or overview*)) or (methodologic* adj3 (review* or overview*))).ti,ab,ot,kf,kw	400809
4	((quantitative adj3 (review* or overview* or synthes*)) or (research adj3 (integrati* or overview*))).ti,ab,ot,kf,kw	19117
5	((integrative adj3 (review* or overview*)) or (collaborative adj3 (review* or overview*)) or (pool* adj3 analy*)).ti,ab,ot,kf,kw	45445
6	(data synthes* or data extraction* or data abstraction*).ti,ab,ot,kf,kw	49585
7	(handsearch* or hand search*).ti,ab,ot,kf,kw	11927
8	(mantel haenszel or peto or der simonian or dersimonian or fixed effect* or latin square*).ti,ab,ot,kf,kw	40602
9	(met analy* or metanaly* or technology assessment* or HTA or HTAs or technology overview* or technology appraisal*).ti,ab,ot,kf,kw	13898
10	(meta regression* or metaregression*).ti,ab,ot,kf,kw	17902
11	(meta-analy* or metaanaly* or systematic review* or biomedical technology assessment* or bio-medical technology assessment*).mp,hw	557173
12	(medline or cochrane or pubmed or medlars or embase or cinahl).ti,ab,hw	415596
13	(cochrane or (health adj2 technology assessment) or evidence report).jw	22391
14	(comparative adj3 (efficacy or effectiveness)).ti,ab,ot,kf,kw	20507
15	(outcomes research or relative effectiveness).ti,ab,ot,kf,kw	12168
16	(intervention adj3 comparison*).ti,ab,ot,kf,kw	5543
17	umbrella review*.ti,ab,ot,kf,kw	2535
18	(metasynthe* or meta-synthe*).ti,ab,ot,kf,kw	2589
19	(evidence adj2 synthesis).ti,ab,ot,kf,kw	10317
20	"review* of reviews".ti,ab,ot,kf,kw	1902
21	scoping review*.ti,ab,ot,kf,kw	34193
22	or/1–21	827095
23	(gp or gps).ti,ab,ot,kf,kw	77269
24	"Family practice"/or "Home care services"/or "Family physicians"/or "Community health services"/or exp "Community health nursing"/or "Community health workers"/or "Preventive health services"/or "general practitioners"/or "physicians, family"/or "physicians, primary care"/or "Public Health Nursing"/	207459
25	("Primary care" or "General practi*" or "Primary health*" or "Community mental health*" or "Family practi*" or "family doctor*" or "Family medicine" or "Family physician*" or "Home care" or "Home based" or "Home health*" or "Community health*" or "Preventive care" or "Prevention program*" or "Preventive service*" or "Preventive health" or "Health promotion").ti,ab,ot,kf,kw	465099
26	exp nurse practitioner/	19653
27	(Nurse practitioner* or community nurs* or community health nurs* or practice nurs* or district nurs* or public health nurs*).ti,ab,ot,kf,kw	38102
28	or/23–27	642571
29	((retain* or retention* or attrition or leave* or leaving or workforce or vacanc* or stay* or positions or turnover or turn over or mobility or return*) adj3 (staff* or personnel or nurs* or doctor* or practitioner* or workforce or worker* or employee*)).ti,ab,ot,kf,kw	60474
30	(staff adj2 (shortage$ or shortfall)).ti,ab,ot,kf,kw	1886
31	career mobility/	12280
32	Job Satisfaction/	30190
33	Health Workforce/	14932
34	(workforce adj2 (plan* or intervention or strateg* or policy or policies or crisis or challenges or program* or model* or forecast* or evaluat*)).ti,ab,ot,kf,kw	4295
35	(staffing adj2 (plan* or intervention or strateg* or policy or policies or crisis or challenges or program* or model* or forecast* or evaluat*)).ti,ab,ot,kf,kw	1981
36	job satisfaction.ti,ab,ot,kf,kw	13273
37	((staff* or employ*) adj2 level).ti,ab,ot,kf,kw	4043
38	retirement/	11648
39	retirement.ti,ab,ot,kf,kw	21380
40	retention.ti,ot,kf,kw	45435
41	pipeline.ti,ab,ot,kf,kw	40303
42	or/29–41	225938
43	28 and 42	18752
44	limit 43 to (english language and yr = "2010 -Current")	12283
45	(editorial or letter or comment).pt	2310174
46	44 not 45	11977
47	22 and 46	886
48	47 and 2023*.dt	109
49	47 and 2024*.dt	132
50	47 and 2025*.dt	20
51	47 and 2022?12?3*.dt	2
52	48 or 49 or 50 or 51	263

Embase < 1974 to 2025 February 05 >

**Table:**

#	Search terms	Records
1	(systematic review or meta-analysis).pt	0
2	meta-analysis/or systematic review/or systematic reviews as topic/or meta-analysis as topic/or "meta analysis (topic)"/or "systematic review (topic)"/or exp technology assessment, biomedical/or network meta-analysis/	730047
3	((systematic* adj3 (review* or overview*)) or (methodologic* adj3 (review* or overview*))).ti,ab,ot,kf,kw	475858
4	((quantitative adj3 (review* or overview* or synthes*)) or (research adj3 (integrati* or overview*))).ti,ab,ot,kf,kw	21566
5	((integrative adj3 (review* or overview*)) or (collaborative adj3 (review* or overview*)) or (pool* adj3 analy*)).ti,ab,ot,kf,kw	62883
6	(data synthes* or data extraction* or data abstraction*).ti,ab,ot,kf,kw	59075
7	(handsearch* or hand search*).ti,ab,ot,kf,kw	14421
8	(mantel haenszel or peto or der simonian or dersimonian or fixed effect* or latin square*).ti,ab,ot,kf,kw	52943
9	(met analy* or metanaly* or technology assessment* or HTA or HTAs or technology overview* or technology appraisal*).ti,ab,ot,kf,kw	23890
10	(meta regression* or metaregression*).ti,ab,ot,kf,kw	21434
11	(meta-analy* or metaanaly* or systematic review* or biomedical technology assessment* or bio-medical technology assessment*).mp,hw	859885
12	(medline or cochrane or pubmed or medlars or embase or cinahl).ti,ab,hw	528956
13	(cochrane or (health adj2 technology assessment) or evidence report).jw	32520
14	(comparative adj3 (efficacy or effectiveness)).ti,ab,ot,kf,kw	29524
15	(outcomes research or relative effectiveness).ti,ab,ot,kf,kw	17338
16	(intervention adj3 comparison*).ti,ab,ot,kf,kw	6793
17	umbrella review*.ti,ab,ot,kf,kw	2590
18	(metasynthe* or meta-synthe*).ti,ab,ot,kf,kw	2771
19	(evidence adj2 synthesis).ti,ab,ot,kf,kw	11354
20	"review* of reviews".ti,ab,ot,kf,kw	2242
21	scoping review*.ti,ab,ot,kf,kw	34899
22	or/1–21	1157788
23	(gp or gps).ti,ab,ot,kf,kw	110234
24	general practice/or home care/or general practitioner/or community care/or exp community health nursing/or health auxiliary/or preventive health service/	384651
25	("Primary care" or "General practi*" or "Primary health*" or "Community mental health*" or "Family practi*" or "family doctor*" or "Family medicine" or "Family physician*" or "Home care" or "Home based" or "Home health*" or "Community health*" or "Preventive care" or "Prevention program*" or "Preventive service*" or "Preventive health" or "Health promotion").ti,ab,ot,kf,kw	584504
26	exp nurse practitioner/	31972
27	(Nurse practitioner* or community nurs* or community health nurs* or practice nurs* or district nurs* or public health nurs*).ti,ab,ot,kf,kw	43719
28	or/23–27	876836
29	((retain* or retention* or attrition or leave* or leaving or workforce or vacanc* or stay* or positions or turnover or turn over or mobility or return*) adj3 (staff* or personnel or nurs* or doctor* or practitioner* or workforce or worker* or employee*)).ti,ab,ot,kf,kw	70988
30	(staff adj2 (shortage$ or shortfall)).ti,ab,ot,kf,kw	2375
31	career mobility/	11341
32	Job Satisfaction/	38080
33	Health Workforce/	14408
34	(workforce adj2 (plan* or intervention or strateg* or policy or policies or crisis or challenges or program* or model* or forecast* or evaluat*)).ti,ab,ot,kf,kw	5073
35	(staffing adj2 (plan* or intervention or strateg* or policy or policies or crisis or challenges or program* or model* or forecast* or evaluat*)).ti,ab,ot,kf,kw	2748
36	job satisfaction.ti,ab,ot,kf,kw	15115
37	((staff* or employ*) adj2 level).ti,ab,ot,kf,kw	5282
38	retirement/	19103
39	retirement.ti,ab,ot,kf,kw	23807
40	retention.ti,ot,kf,kw	52010
41	pipeline.ti,ab,ot,kf,kw	55575
42	or/29–41	266756
43	28 and 42	23215
44	limit 43 to (english language and yr = "2010 -Current")	16363
45	(editorial or letter or comment).pt	2155020
46	44 not 45	15886
47	22 and 46	1104
48	47 and 2025*.dc	17
49	47 and 2024*.dc	183
50	47 and 2023*.dc	116
51	47 and 2023123*.dc	1
52	48 or 49 or 50 or 51	

HMIC Health Management Information Consortium < 1979 to November 2024 >

**Table:**

#	Search terms	Records
1	meta analysis/	789
2	systematic reviews/	3473
3	health technology assessment/	610
4	((systematic* adj3 (review* or overview*)) or (methodologic* adj3 (review* or overview*))).mp	5528
5	((quantitative adj3 (review* or overview* or synthes*)) or (research adj3 (integrati* or overview*))).mp	398
6	((integrative adj3 (review* or overview*)) or (collaborative adj3 (review* or overview*)) or (pool* adj3 analy*)).mp	357
7	(data synthes* or data extraction* or data abstraction*).mp	608
8	(handsearch* or hand search*).mp	307
9	(mantel haenszel or peto or der simonian or dersimonian or fixed effect* or latin square*).mp	404
10	(met analy* or metanaly* or technology assessment* or HTA or HTAs or technology overview* or technology appraisal*).mp	1308
11	(meta regression* or metaregression*).mp	139
12	(meta-analy* or metaanaly* or systematic review* or biomedical technology assessment* or bio-medical technology assessment*).mp	5907
13	(medline or cochrane or pubmed or medlars or embase or cinahl).mp	3300
14	(cochrane or (health adj2 technology assessment) or evidence report).jw	1117
15	(comparative adj3 (efficacy or effectiveness)).mp	144
16	(outcomes research or relative effectiveness).mp	209
17	(intervention adj3 comparison*).mp	86
18	umbrella review*.mp	26
19	(metasynthe* or meta-synthe*).mp	78
20	(evidence adj2 synthesis).mp	182
21	"review* of reviews".mp	85
22	scoping review*.mp	458
23	or/1–22	10353
24	(gp or gps).mp	13823
25	general practice/	9044
26	primary care/	12905
27	primary care nursing/	70
28	ambulatory care/	258
29	home care/	2796
30	"home care of patient"/	740
31	exp general practitioners/	10531
32	community health services/	1901
33	district nurse services/	66
34	family planning services/	70
35	health centres/	390
36	immunisation services/	254
37	screening services/	120
38	community health care/	1683
39	community nursing/	1213
40	community services/	310
41	family health services/	113
42	Community health workers/	63
43	preventive medicine/	2053
44	("Primary care" or "General practi*" or "Primary health*" or "Community mental health*" or "Family practi*" or "family doctor*" or "Family medicine" or "Family physician*" or "Home care" or "Home based" or "Home health*" or "Community health*" or "Preventive care" or "Prevention program*" or "Preventive service*" or "Preventive health" or "Health promotion").mp	63819
45	exp nurse practitioners/	788
46	(Nurse practitioner* or community nurs* or community health nurs* or practice nurs* or district nurs* or public health nurs*).mp	5939
47	or/24–46	70511
48	23 and 47	1870
49	((retain* or retention* or attrition or leave* or leaving or workforce or vacanc* or stay* or positions or turnover or turn over or mobility or return*) adj3 (staff* or personnel or nurs* or doctor* or practitioner* or workforce or worker* or employee*)).mp	12936
50	(staff adj2 (shortage$ or shortfall)).mp	1289
51	exp career development/	1450
52	job satisfaction/	1195
53	human resources management/	1754
54	job enlargement/	3
55	staff morale/	322
56	staff surveys/	338
57	exp human resources/	74436
58	workforce/or human resources/or labour supply/or labour/or manpower services commission/or staff/or workers/or workforce planning/	9410
59	exp labour supply/	9146
60	manpower services commission/	15
61	workforce planning/	1649
62	(workforce adj2 (plan* or intervention or strateg* or policy or policies or crisis or challenges or program* or model* or forecast* or evaluat*)).mp	2218
63	(staffing adj2 (plan* or intervention or strateg* or policy or policies or crisis or challenges or program* or model* or forecast* or evaluat*)).mp	203
64	job satisfaction.mp	1816
65	((staff* or employ*) adj2 level).mp	173
66	exp Retirement/	959
67	retirement.mp	2681
68	retention.mp	2999
69	pipeline.mp	126
70	or/49–69	82788
71	48 and 70	377
72	limit 71 to yr = "2010 -Current"	209
73	72 and 2025*.up	0
74	72 and 2024*.up	7
75	72 and 2023*.up	18
76	72 and 2022123*.up	0
77	73 or 74 or 75 or 76	

## CINAHL Complete

((PT "systematic review") OR (PT meta-analysis)) OR (MH "systematic review") OR ((TI review*) OR (AB “systematic review*”) OR (TI “systematic overview*” OR AB “systematic overview*”) OR (TI “evidence synthes*” OR AB “evidence synthes*”) OR (TI "data synthes*" OR AB "data synthes*") OR (TI "data extraction*" OR AB "data extraction*") OR (TI "data abstraction*" OR AB "data abstraction*") OR (TI handsearch* OR AB handsearch*) OR (TI "hand search*" OR AB "hand search*") OR ((TI "mantel haenszel" OR AB "mantel haenszel") OR (TI peto OR AB peto) OR (TI "der simonian" OR AB "der simonian") OR (TI dersimonian OR AB dersimonian) OR (TI "fixed effect*" OR AB "fixed effect*") OR (TI "latin square*" OR AB "latin square*") OR (TI "meta analy*" OR AB "meta analy*") OR (TI metaanaly* OR AB metaanaly*) OR (TI "technology assessment*" OR AB "technology assessment*") OR (TI HTA OR AB HTA) OR (TI HTAs OR AB HTAs) OR (TI "technology overview*" OR AB "technology overview*") OR (TI "technology appraisal*" OR AB "technology appraisal*") OR (TI "meta regression*" OR AB "meta regression*") OR (TI metaregression* OR AB metaregression*) OR (TI medline OR AB medline) OR (TI cochrane OR AB cochrane) OR (TI pubmed OR AB pubmed) OR (TI medlars OR AB medlars) OR (TI embase OR AB embase) OR (TI cinahl OR AB cinahl) OR (AB "umbrella review*") OR (TI metasynthe* OR AB metasynthe*) OR (TI meta-synthe* OR AB meta-synthe*) OR (AB "review of reviews") OR (AB "scoping review*")).

## AND

((TI gp OR AB gp) OR (TI gps OR AB gps) OR (MH "Primary Health Care") OR (MH "Family practice") OR (MH "Community health services") OR (MH "Family Nurse Practitioners") OR (MH "Community health nursing + ") OR (MH "Community health workers") OR (MH "physicians, family") OR (TI "Primary care" OR AB "Primary care") OR (TI "General practi*" OR AB "General practi*") OR (TI "Primary health*" OR AB "Primary health*") OR (TI "Community mental health*" OR AB "Community mental health*") OR (TI "Family practi*" OR AB "Family practi*") OR (TI "family doctor*" OR AB "family doctor*") OR (TI "Family medicine" OR AB "Family medicine") OR (TI "Family physician*" OR AB "Family physician*") OR (TI "Nurse practitioner*" OR AB "Nurse practitioner*") OR (TI "community nurs*" OR AB "community nurs*") OR (TI "community health nurs*" OR AB "community health nurs*") OR (TI "practice nurs*" OR AB "practice nurs*") OR (TI "district nurs*" OR AB "district nurs*") OR (TI "public health nurs*" OR AB "public health nurs*")).

## AND

(((TI retain* OR AB retain*) OR (TI retention* OR AB retention*) OR (TI attrition OR AB attrition) OR (TI leave* OR AB leave*) OR (TI leaving OR AB leaving) OR (TI workforce OR AB workforce) OR (TI vacanc* OR AB vacanc*) OR (TI stay*) OR (TI positions) OR (TI turnover OR AB turnover) OR (TI "turn over" OR AB "turn over") OR (TI career OR AB “career mobility” OR AB “career progession”) OR (TI staff* OR AB “staff* level*”) OR (TI personnel) OR (TI employee* OR AB employee*) OR (TI pipeline OR AB pipeline) OR (TI "job satisfaction" OR AB "job satisfaction") OR (TI "retirement" OR AB "retirement"))) OR (MH "Personnel Retention") OR (MH "Personnel Shortage") OR (MH retirement) OR (MH "career mobility").

Limit to publication date 2015 onwards.

## British Nursing Index (BNI)

Noft(“systematic review*” OR “systematic overview*” OR “evidence synthes*” OR "data synthes*" OR "data extraction*" OR "data abstraction*" OR handsearch* OR "hand search*" OR "mantel haenszel" OR peto OR "der simonian" OR dersimonian OR "fixed effect*" OR "latin square*" OR "meta analy*" OR metaanaly* OR "technology assessment*" OR HTA OR HTAs OR "technology overview*” OR "technology appraisal*" OR "meta regression*" OR metaregression* OR medline OR cochrane OR pubmed OR medlars OR embase OR cinahl OR "umbrella review*" OR metasynthe* OR meta-synthe* OR "review of reviews" OR "scoping review*").

## AND

Noft(gp OR gps OR "Family practice" OR "Community health services" OR “Community health nursing" OR "Community health workers" OR "physicians, family" OR "Primary care" OR "General practi*" OR "Primary health*" OR "Community mental health*" OR "Family practi*" OR "family doctor” OR "Family medicine" OR "Family physician*" OR "Nurse practitioner*" OR "community nurs*" OR "community health nurs*" OR "practice nurs*" OR "district nurs*" OR "public health nurs*").

## AND

noft(Retain OR retention* OR attrition OR leave* OR leaving OR workforce OR vacanc* OR turnover OR "turn over" OR career OR “staff* level*” OR “staff shortage*” OR “staff plan*” OR employee* OR pipeline OR "job satisfaction" OR retirement).

Limit to publication date 2015 onwards.
